# Lithium effects on impulsivity and emotional processing

**DOI:** 10.1038/s41598-025-29216-7

**Published:** 2025-11-23

**Authors:** Fitri Fareez Ramli, Catherine J. Harmer, Philip J. Cowen, Beata R. Godlewska

**Affiliations:** 1 Clinical Psychopharmacology Group, Department of Psychiatry, University of Oxford, Warneford Hospital, Oxford, OX3 7JX UK; 2 Oxford Health NHS Foundation Trust, Oxford, UK; 3 Department of Pharmacology, Faculty of Medicine, Universiti Kebangsaan Malaysia, Kuala Lumpur, 56000 Malaysia

**Keywords:** Emotional processing, Impulsivity, Lithium, Reward, Psychology, Clinical pharmacology, Translational research

## Abstract

Lithium is an effective treatment for mood disorders. However, its mechanism of action concerning its effect on impulsivity and emotional processing is still unclear. The current study aimed to investigate the effect of a 5-day lithium treatment on decision-making, reward-seeking and emotional processing in healthy volunteers. We conducted a double-blind, placebo-controlled, cross-over design study involving sixteen participants aged 18–50 years. Participants received lithium 800 mg once daily followed by matching placebo or vice versa for five days in a random order. Impulsivity and emotional processing were assessed on day six using the Cambridge Gambling Task and the Emotional Testing Battery, respectively. There were significant interactions with large effect sizes between treatment and order for delay aversion (F_1,14_ = 13.79, *p* = 0.002, partial η^2^ = 0.496) and reward-seeking (F_1,14_ = 34.065, p = < 0.001, partial η^2^ = 0.709), but the post-hoc tests suggested only moderate, inconclusive effects at either visit. There was a significant interaction between treatment, emotion and order with a large effect size for the facial expression recognition task. The post-hoc analyses found that during the first visit, relative to placebo, lithium-treated participants showed a higher accuracy in recognising disgust and lower misclassification rates and response bias in recognising sad facial expressions, but higher accuracy and response bias in recognising fear during the second visit. We found a significant interaction between treatment and valence with a large effect size for emotional encoding of self-referent words. Post-hoc analysis showed that lithium was associated with a longer reaction time to encode negative self-referent words than placebo. Short-term lithium treatment in healthy participants produced a positive emotional bias in facial expression recognition and emotional encoding of self-referent words, but no significant effects on impulsivity and reward seeking. The early induction of positive bias in emotional processing may contribute to lithium’s effectiveness in mood disorders.

**Supplementary Information:**

The online version contains supplementary material available at 10.1038/s41598-025-29216-7.

## Introduction

Lithium remains the primary prophylactic option to reduce recurrences of manic and depressive episodes in patients with bipolar disorder^1^. Also, lithium has additional benefits in acute phase treatments of both manic and depressive states as well as in the presence of other comorbidities (substance abuse, obsessive-compulsive symptoms and neurological deficits)^2^. The effectiveness of lithium also extends to another mood disorder, unipolar depression, in which lithium has been reported to be effective in treatment-resistant depression with added benefits in reducing suicide and mortality^3–5^.

The modulating effects of lithium in suicide prevention might be attributable to its effect in lowering impulsivity^6,7^. Acute and chronic lithium treatments have been shown to reduce both motor and cognitive impulsivity in preclinical models^8–10^. In clinical populations, randomised, double-blind, placebo-controlled treatment studies showed that lithium reduced impulsivity in people with chronic impulsive aggressive behaviour, medically severe suicide attempters and pathological gamblers with bipolar spectrum disorders^6,11,12^. However, the assessment of lithium’s effects on impulsivity in these populations might be confounded by disease states, heterogeneity of psychiatric disorders within a study population and reliance on the self-reported measures of impulsivity. Therefore, a laboratory measure of impulsivity in healthy volunteers may shed light on the mechanism of action of lithium in the absence of those confounding factors.

The comment on causation referred to this section: “lithium was reported to reduce impulsivity in people with chronic impulsive aggressive behaviour, medically severe suicide attempters and pathological gamblers with bipolar spectrum disorders“. If these aren’t causal studies i.e. where lithium was manipulated via randomization, I don’t think they should be reported as showing that lithium reduces - whatever the authors of those papers report. Rather that treated patients showed reduced impulsivity relative to untreated, or similar. These type of studies are too confounded usually to be much use causally.

Apart from impulsivity, lithium also reduces depressive symptoms, relapse frequency and hospitalisation in mood disorders, potentially through its regulating effects on emotion^2,4,5,13^. Although mood is more pervasive and persistent phenomenon than emotion (which is more targeted and short-lived), changes in emotion can have a prominent impact on mood. For instance, patients with bipolar disorder exhibit positive correlations between emotional dysregulation and both manic and depressive symptoms. Interestingly, the association with depressive symptoms appears stronger than that with manic symptoms^14^.

Lithium can regulate emotion through activations (and deactivations) and alterations of connectivity in specific brain regions, including fronto-limbic networks^15^. However, studies assessing lithium’s mechanism of action on other aspects of emotion are still limited. One potential area is emotional processing, which can be influenced by lithium’s antidepressant effects in bipolar and unipolar depression^5,16^. Emotional processing is proposed as a direct antidepressant target and is a putative surrogate marker of antidepressant potential^17^. Another interesting area is reward processing, which can be measured using the Cambridge Gambling Task. Lithium has been shown to normalise response to reward processing through its effects on reducing outcome-related activities in the dorsolateral prefrontal cortex in patients with bipolar disorder outside their major mood episodes that required immediate treatment^18^. To our knowledge, the evidence of lithium’s effect on decision-making, reward-seeking behaviour and emotional processing is limited in the literature.

The current study initially aimed to investigate the effect of a 5-day lithium vs. placebo treatment on decision-making, reward-seeking and emotional processing in healthy volunteers using a cross-over design study. However, due to significant order effects observed in many parameters, we were only able to investigate the differences between treatments in each visit separately in the post-hoc analysis.

We hypothesized that lithium treatment would be associated with lower impulsivity, lower reward-seeking and positive biases in emotional processing in healthy volunteers, consistent with our previous findings using the lithium-mimetic agent ebselen^19^.

## Methods

We conducted a double-blind, placebo-controlled, cross-over design study. We included participants aged 18–50 years with normal thyroid and renal function. The study received ethical approval from the Medical Sciences Interdivisional Research Ethics Committee (IDREC), University of Oxford (R82462/RE002) and was performed in accordance with the Declaration of Helsinki. All methods were performed in accordance with the relevant guidelines and regulations. Informed consent was obtained from all participants before the study. The study was registered on the Open Science Framework Registries on the 26th July 2024, registration link 10.17605/OSF.IO/W2MXQ. During the screening session, participants were interviewed using the Structured Clinical Interview for Diagnostic and Statistical Manual-5^20^. Venous blood was withdrawn to measure levels of thyroid-stimulating hormone and kidney function. Urine was tested for pregnancy in female participants. Participants were required to use effective contraception from the screening visit until 30 days after completing the study medication treatment.

Our exclusion criteria were any history or current psychiatric disorders; history of regular illicit drug use or any use within the previous three months; history of or current general medical conditions that, in the opinion of the investigator, could interfere with the safety of the participant or the scientific integrity of the study; history of cardiac diseases (abnormal rhythm or conduction defect), Addison’s disease or diabetes insipidus; history of hypersensitivity reaction towards lithium or components of placebo/capsules; current pregnancy, breastfeeding, or planning a pregnancy; and planned medical treatment within the study period that might interfere with the study procedures. Participants who participated in a previous study involving the same or similar decision-making and reward-seeking behaviour-processing tasks were included if the duration between studies was more than three months. Based on our previous study with ebselen^19^, the sample size to detect a 1.6% difference in accuracy for recognising happy facial expressions, with a significance level of 0.05 and power of 0.9, was 11 participants.

**Fig. 1:**
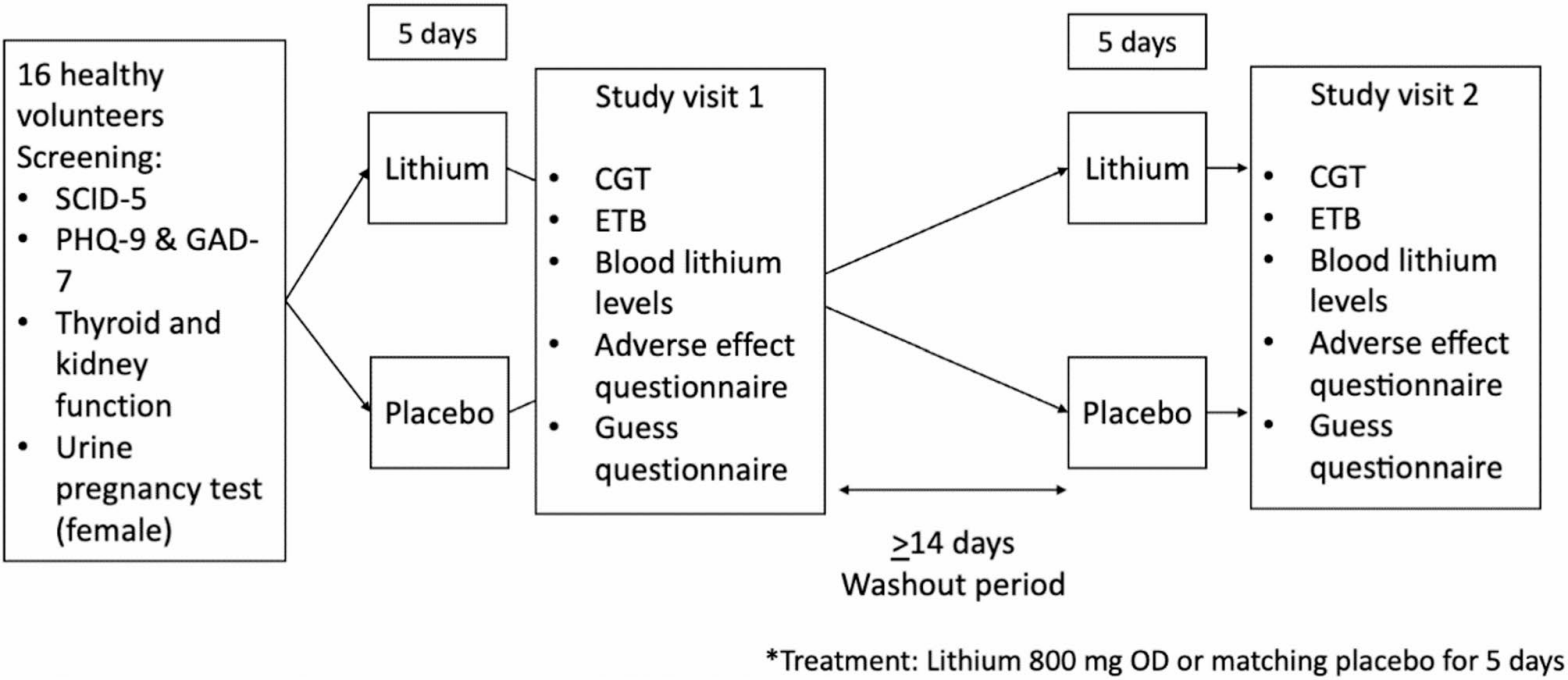
A flowchart of the study design.

Participants were also required to complete the Patient Health Questionnaire-9 (PHQ-9) and the Generalized Anxiety Disorder-7 (GAD-7)^21,22^. Eligible participants were then randomized to receive lithium 800 mg once daily at night for five days followed by matching placebo for a similar duration or vice versa in a random order. A washout period (drug-free) between treatments was at least two weeks. Both participants and researchers involved directly with participants’ screening and testing were blinded to treatment allocation. Participants were tested after five days (on day six) of each treatment using the Cambridge Gambling Task (CGT) and the Emotional Testing Battery (ETB). Other procedures included venous blood taking to assess lithium levels, a treatment guess questionnaire and an adverse effect questionnaire. A flowchart of the study design is presented in Fig. 1. For the treatment guess, at the end of each study visit, participants were asked to guess whether they had received lithium or placebo. We did not include the subjective mood measures in our analysis, as participants were healthy volunteers without mood disorders (screened by SCID-5, PHQ-9, and GAD-7). In this context, we anticipated that lithium would have negligible effects on self-reported mood. Instead, we focussed on emotional processing measures, as shifts in emotional bias are considered a more sensitive and direct pharmacological effect of drug in the cognitive neuropsychological model of antidepressant drug action^17^. This battery allows detection of subtle changes in emotional processing that may precede changes in subjective mood.

### Cambridge gambling task (CGT)

The Cambridge Gambling Task (CGT) assesses decision-making and reward-seeking behaviour outside a learning context^23^. An audio instruction was given before the task started. The participant was informed that a yellow token was hidden in one of the ten boxes located at the top of the screen. The boxes were either red or blue, with varying ratios (1:9, 8:2, 7:3, 6:4, or 5:5 and vice versa). The first step involved the colour selection (red or blue), in which the yellow token might be hidden. The bet selection was the next step in which proportions of 5%, 25%, 50%, 75% and 95% of the total points were presented in the middle of the screen. In each trial, the proportions either increased (ascending order) or decreased (descending order) accordingly, and each proportion was shown for five seconds. The participants were instructed to select the bet based on their certainty. Also, participants were informed to accumulate as many points as possible. The result would appear immediately on the screen when the bet was selected. If the response was correct, the bet amount was added to the accumulated box. However, the incorrect response might result in the deduction of the accumulated points from the bet made by the participant. In case when no bet was selected, the last bet presented was automatically selected. No monetary incentives were given to the participants for the accumulated points.

The parameters measured in the CGT included delay aversion or impulsivity, reward-seeking, deliberation time, risk adjustment and quality of decision-making. The quality of decision-making was measured by counting the proportion of the trials where the participants selected a colour with more boxes. The average duration of choosing the more likely outcome was also measured as a part of the quality of decision-making. The time taken for the participant to select the bet amount, regardless of the quality of decision-making, was the deliberation time. Impulsivity or delay aversion was measured by counting the difference between the average bet from the ascending order and the descending order trials in trials in which the optimal outcome was selected. Reward-seeking referred to the average proportion of the current points that the participants were willing to bet with the more likely outcome. Risk adjustment was measured from the proportions of points the participants opted to bet by considering the more likely outcome.

### Emotional testing battery (ETB)

The battery consists of five tasks, including the Facial Expression Recognition Task (FERT), the Emotional Categorization Task (ECAT), the Emotional Recall Task (EREC), the Facial Dot-Probe Task (F-DOT) and the Emotional Recognition Memory Task (EMEM)^24,25^. Different versions of each task (FERT, ECAT and EMEM) were used to minimize practice effects. The FERT was the first task conducted in which participants were presented with one face of different emotion (40 for each emotion, ten neutral faces) and intensities (0–100% with a 10% increment) at a time for 500 ms. Participants were instructed to press the button on the keyboards immediately to indicate which emotion was presented (sad, fear, happy, surprise, disgust, anger or neutral). The ECAT was the second task in which participants were presented with one self-referent word at a time and were instructed to choose the valence of the words (positive or negative).

The third task was the FDOT, in which participants were initially presented with a plus sign before two faces (one neutral and another emotional (fear or happy)) arranged vertically. As soon as the faces disappeared, two dots arranged either horizontally or vertically would appear at the top or the bottom of the faces. Participants were instructed to indicate the orientation of the dots by pressing the button (: or.) on the keyword. The EREC was the task conducted after the FDOT when participants were asked to recall as many words as possible and write them down on the paper within four minutes. The EMEM was the last task when participants were presented again the self-referent words, and were asked to indicate whether the words were presented in the ECAT task or not (responded as ‘yes’ or ‘no’).

The parameters of interest included accuracy (FERT, ECAT, EREC, EMEM), reaction time (RT) (FERT, ECAT, EMEM), misclassification (FERT, EREC, EMEM), measure of response bias (beta) and signal detection (d prime) for FERT and EMEM and attentional vigilance score for the FDOT. D prime (d’) is a measure of signal detection, with higher values reflecting greater sensitivity in distinguishing a signal from noise. For instance, in the FERT, a d prime value of 0.9 for happy facial expressions indicates relatively higher sensitivity. In this case, participants are more accurate in correctly identifying happy faces as happy rather than misclassifying as other emotions. Beta value is a measure of response bias, with higher values reflecting a more conservative response styles. For example, in the FERT, a beta value of 0.9 for happy facial expressions reflects a relatively more conservative response style. Meaning that participants are more likely to label happy faces as happy only when they are confident that the faces presented truly represents happy expressions.

Signal detection (d prime) indices were calculated for each emotion using the following formula: d prime = 0.5 + ((y – x)(1 + y – x)/4y (1 – x)), 0 < d’ < 1). Response bias (beta) was calculated as: beta = y(1 – y) – x(1 – x)/y(1 – y) + x(1 – x), − 1 < ß < 1 (x is a false alarm (proportion of incorrect responses), and y is a hit rate (proportion of correct responses). In our analysis, we treated each emotion as a binary outcome (correct or incorrect) to compute d prime and beta values, providing general measures across emotions. However, it does not differentiate between specific patterns of misclassification. We acknowledged this as a limitation and consider a confusion matrix-based approach to be a valuable direction for future work, particularly with a larger sample size.

### Statistical analysis

Continuous variables are presented in means and standard error of means. Categorical variables are presented in numbers and proportions. Analysis of variance (ANOVA) was used to analyse the interactions between treatment (within-subject) and order (between-subject) for CGT parameters. Additional within-subject factors, including valence (positive vs. negative) or emotion (sad, fear, happy, surprise, disgust, anger and neutral) and condition (for FDOT only; masked vs. unmasked), were included in ANOVA for ETB parameters. As there were significant interactions between order and treatment in various parameters, we decided to conduct post-hoc independent *t*-tests in the first visit and the second visit separately. The only exception was the reaction time for encoding self-referent words, as there was no significant order interaction. Therefore, we analysed this measure using a paired *t*-test to compare between treatments.

We noticed that several data distributions were skewed, as expected with a small sample size. However, both t-tests and ANOVAs are generally considered robust to moderate deviations from normality, particularly with balanced designs. To address potential violations, we inspected distributions, verified equality of variances using Levene’s test for t-tests, and for repeated-measures ANOVAs, we applied Greenhouse–Geisser corrections when the assumption of sphericity was violated. Additionally, we conducted supplementary analysis on our proportion data (only for data with a range between 0 and 1) using an arcsine square-root transformation.

Given the small sample size, we placed greater emphasis on effect sizes partial η^2^ for ANOVAs (small: 0.01, moderate: 0.06, and large: 0.14) as indicators of the magnitude and the potential relevance of the outcomes. To further explore the source of interaction effects in the ANOVA, post-hoc tests were performed when either the p-value was < 0.05 or the effect size reached at least a moderate level (partial η^2^ > 0.06). For post-hoc t-tests, we referred to Cohen’s *d* (small: 0.2, moderate: 0.5, large: 0.8) and its confidence interval to determine the presence of a statistically meaningful effect.

## Results

Sixteen participants (11 females, 5 males) aged 19–50 years (27.5 *±* 1.7 years) were included in the final analysis. The means and standard error of means for PHQ-9 and GAD-7 were 1.1 *±* 0.4 and 1.6 *±* 0.5, respectively, corresponding to no/minimal depression/anxiety^21,22^. There were no significant differences between lithium and placebo in adverse effects of nausea, diarrhoea, thirst, excessive urination, tremors, dry mouth and metallic taste. There were no significant differences in treatment guess (χ^2^(1) = 1.607, *p* = 0.448), suggesting that there was no clear evidence of unblinding. The blood levels of lithium during treatment with lithium were 0.44 *±* 0.05 mmol/l (0.3–0.9 mmol/l). As expected, lithium levels during the placebo treatment were negligible. The washout period ranges between 14 and 32 days.

### Cambridge gambling tasks

Significant interactions between treatment and order with large effect sizes were observed for delay aversion (F_1,14_ = 13.79, *p* = 0.002, partial η^2^ = 0.496) and reward-seeking (arcsine transformed: F_1,14_ = 34.065, p = < 0.001, partial η^2^ = 0.709). There was a moderate effect size for the interactions between treatment and order in risk adjustment (F_1,14_ = 0.893, *p* = 0.361, partial η^2^ = 0.060) and small effect sizes for the interactions between treatment and order in quality of decision-making (arcsine transformed: F_1,14_ = 0.134, *p* = 0.720, partial η^2^ = 0.009) and deliberation time (F_1,14_ = 0.187, *p* = 0.672, partial η^2^ = 0.013). However, no significant interactions were observed for these parameters.

The post-hoc independent *t*-tests for CGT parameters of delay aversion (Cohen’s d = 0.594, 95%CI: −0.420–1.588) and reward-seeking (arcsine transformed: Cohen’s d = −0.662, 95%CI: −1.661–0.358) reported moderate effect sizes confidence intervals included zero (Table S1) for the first visit, suggesting that these effects were not statistically reliable and should be interpreted with caution. Similarly, moderate effect sizes with confidence intervals included zero were observed for delay aversion (Cohen’s d = −0.574 95%CI: −1.567–0.438) and reward-seeking (arcsine transformed: Cohen’s d = −0.541, 95%CI: −1.532–0.468) for the second visit, indicating that these effects were not statistically reliable.

As we hypothesize that lithium’s potential anti-suicidal effects may be mediated through reductions in impulsivity, we conducted additional analyses to examine the correlation between lithium levels and delay aversion, a lab measure of impulsivity. We found no significant correlation between lithium levels and delay aversion (*r*(10) = 0.12, *p* = 0.71, Figure S1) during the lithium treatment.

### Emotional testing battery

There was a significant three-way interaction with a large effect size between treatment, valence and order for the FERT accuracy (arcsine transformed: F_3,41_ =6.670, p = < 0.001, partial η^2^ = 0.323). There was a large effect size but no significant two-way interaction between treatment and order (arcsine transformed: F_1,14_ = 1.769, *p* = 0.205, partial η^2^ = 0.112). However, there were no significant two-way interactions between emotion and order (arcsine transformed: F_3,37_ = 0.606, *p* = 0.595, partial η^2^ = 0.042) and treatment and emotion (arcsine transformed: F_3,41_ = 1.041, *p* = 0.384, partial η^2^ = 0.069), both only showing small-to-moderate effect sizes. A post-hoc independent *t*-test (arcsine transformed: *t*(14) = 2.516, 95%CI: 0.015–0.194, *p* = 0.025, Cohen’s *d* = 1.258, 95%CI: 0.157–2.323) revealed higher accuracy in recognising disgust facial expression under lithium (0.81 *±* 0.03) compared to placebo (0.71 *±* 0.03) during the first visit. The large effect size and a confidence interval that did not include zero indicate a statistically reliable effect. Besides, there were small-to-moderate effect sizes with confidence intervals that included zero in recognising other emotions between treatment in the first visit (Table S2). Also, effect size estimates for FERT accuracy for any emotion during the second visit ranged from small to moderate with all confidence intervals included zero, indicating that these effects were not statistically reliable.

There was a significant three-way ANOVA with a large effect size (arcsine transformed: F_6,84_ = 8.432, p = < 0.001, partial η^2^ = 0.376) for the FERT misclassifications between treatment, order and emotion. There were small-to-moderate effect sizes for two-way interactions between treatment and order (arcsine transformed: F_1,14_ = 0.412 *p* = 0.531, partial η^2^ = 0.029) and treatment and emotion (arcsine transformed: F_6,84_ = 0.990, *p* = 0.438, partial η^2^ = 0.066) and a large effect size for the interaction between emotion and order (arcsine transformed: F_6,84_ = 1.748, *p* = 0.120, partial η^2^ = 0.111), but these two-way interactions were not statistically significant. A follow-up independent *t*-test found that the lithium group (0.21 *±* 0.01) had a lower misclassification rate of sad facial expressions compared to the placebo group (0.28 *±* 0.03) during the first visit (arcsine transformed: *t*(14) = −2.204, 95%CI: −0.142 – −0.002, *p* = 0.045, Cohen’s *d* = −1.102, 95%CI: (−2.146 – −0.025). The large effect size, together with a confidence interval that did not include zero, indicates a statistically reliable effect. Effect size estimates for other emotions during the first visit ranged from small to moderate (Table S2), but all confidence intervals included zero, indicating that the effects were not statistically reliable. Similarly, effect size estimates for emotions (except for fear) during the second visit ranged from small to moderate-to-large (Table S2), but all confidence intervals included zero. For fear emotion, we observed a higher misclassification in the lithium group (0.22 *±* 0.03) compared to the placebo (0.12 *±* 0.03). The large effect size with a confidence interval that did not cross zero (arcsine transformed: *t*(14) = 2.512, 95%CI: 0.015–0.186, *p* = 0.025, Cohen’s *d* = 1.256, 95%CI: (−0.155–2.321) indicates a statistically reliable effect.

There was a significant three-way ANOVA with a large effect size (F_6,78_ = 3.655, *p* = 0.003, partial η^2^ = 0.219) for the FERT d prime. There was a large effect size in two-way ANOVAs between treatment and order (F_1,13_ = 2.727, *p* = 0.123, partial η^2^ = 0.173) and moderate effect sizes for emotion x order (F_6,78_ = 0.489, *p* = 0.815, partial η^2^ = 0.036) and treatment x emotion (F_6,78_ = 0.629, *p* = 0.707, partial η^2^ = 0.046), but all corresponding p-values were > 0.05. Post-hoc independent *t*-tests demonstrated effect size estimates ranged from negligible to very large for the first visit and negligible to small-to-moderate for the second visit with all confidence intervals crossing zero (Table S2), indicating statistically unreliable effects.

There was a significant three-way ANOVA with a large effect size (F_2,24_ = 9.901, p = < 0.001, partial η^2^ = 0.432) for the FERT beta. There was a significant two-way interaction with a large effect size between treatment and order (F_1,13_ = 4.832, *p* = 0.047, partial η^2^ = 0.271). In contrast, no significant interactions were observed between emotion and order (F_2,29_ = 0.581, *p* = 0.583, partial η^2^ = 0.043) or treatment and emotion (F_2,24_ = 1.415, *p* = 0.262, partial η^2^ = 0.098), although both showed moderate effect sizes. A follow-up independent *t*-test found that the lithium group (0.69 *±* 0.03) had higher beta values for sadness compared to placebo (0.48 *±* 0.09) with a large effect size during the first session (*t*(9.06) = 2.241, 95%CI: −0.002–0.428, *p* = 0.052, Cohen’s *d* = 1.121, 95%CI: 0.041–2.166). Negligible to moderate-to-large effect sizes were observed for beta values for other emotions but all corresponding confidence intervals crossed zero (Table S2). In the second session, the lithium group (0.66 *±* 0.06) had lower beta values for fear than the placebo (0.86 *±* 0.05) group. The large effect size with a confidence interval that did not include zero indicate a statistically reliable effect (*t*(14) = −2.576, 95%CI: −0.372 – −0.034, *p* = 0.022, Cohen’s *d* = −1.288, 95%CI: (−2.357 – −0.181). A lower beta value in the lithium group indicates a less conservative response towards fear facial expressions. In other words, participants receiving lithium were more likely to identify faces as fearful, even when they were uncertain.

**Fig. 2:**
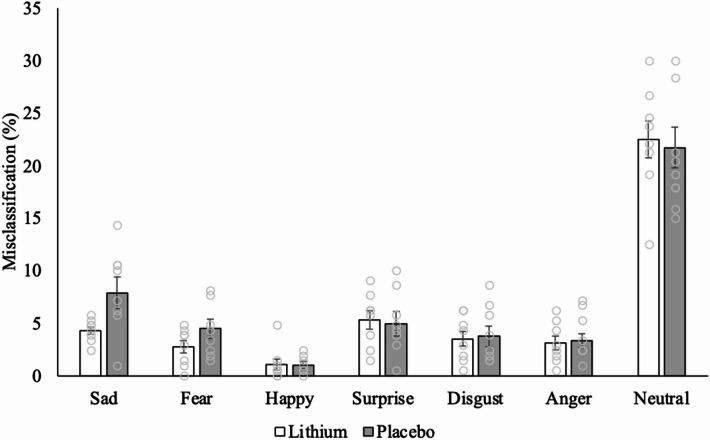
Means (± SEMs) reaction time in the ECAT task. There was a two-way interaction between treatment and valence (F_1,14_ = 4.630, *p* = 0.049, partial η^2^ = 0.249). A follow-up paired *t*-test (*t*(15) = 3.115, 95%CI: 0.052–0.279, *p* = 0.007, Cohen’s *d* = 0.779) showed that lithium treatment significantly prolonged the response time to negative self-referent words compared to placebo treatment.

The effect size for the interaction between treatment, valance, and order was observed in the ECAT reaction time (RT) (F_1,14_ = 0.415, *p* = 0.530, partial η^2^ = 0.029) was small-to-moderate effect but the result did not reach a statistical significance. There was a significant two-way interaction between treatment and valence with a large effect size (F_1,14_ = 4.630, *p* = 0.049, partial η^2^ = 0.249). A follow-up paired *t*-test (*t*(15) = 3.115, 95%CI: 0.052–0.279, *p* = 0.007, Cohen’s *d* = 0.779, 95%CI: 0.207–1.331) showed that lithium treatment (1.149 *±* 0.081 s) significantly increased the response time to negative self-referent words compared to placebo treatment (0.983 *±* 0.066 s) with a large effect size (Fig. 2). In contrast, a small-to-moderate effect size was observed between lithium (1.006 *±* 0.063 s) and placebo (0.923 *±* 0.048 s) treatment in the response time to positive self-referent words (*t*(15) = 1.409, 95%CI: −0.042–0.208, *p* = 0.179, Cohen’s *d* = 0.352, 95%CI: −0.159–0.852). However, the confidence interval included zero, indicating that this effect was not statistically reliable. The effect sizes for ECAT accuracy ranged from small to small-to-moderate for both three-way and two-way interactions (Table S3). The effect sizes for ECAT accuracy were small-to-moderate for both valences across both visits, with all confidence intervals including zero (Table S2), indicating that these effects were not statistically reliable.

There was a significant three-way interaction between treatment, valance and order with a large effect size for the EREC accuracy (F_1,14_ = 7.710, *p* = 0.015, partial η^2^ = 0.355). However, the effect sizes were negligible and no significant two-way interactions were found between treatment and valence (F_1,14_ = 0.006, *p* = 0.941, partial η^2^ = 0.000), valence and order (F_1,14_ = 0.009, *p* = 0.927, partial η^2^ = 0.001) and treatment and order (F_1,14_ = 0.072, *p* = 0.792, partial η^2^ = 0.005). Follow-up independent *t*-tests showed small-to-moderate effect sizes for both positive and negative valences at both visits, with all confidence intervals including zero (Table S2). For EREC misclassification, both three-way and two-way interactions showed small effect sizes, except for the treatment × valence interaction, which demonstrated a moderate effect size (Table S3). Post-hoc independent *t*-tests indicated large effect size estimates for both valences and visits. However, all confidence intervals crossed zero (Table S2), suggesting that these effect sizes were not statistically reliable.

The effect sizes ranged from small to large with significant interactions for three-way and various two-way interactions for EMEM (accuracy, RT, misclassification, d prime, beta) and FDOT (attentional vigilance score) (Table S3). However, all confidence intervals crossed zero (Table S2), suggesting that these effect sizes were not statistically reliable.

## Discussion

We found that lithium was associated with a longer reaction time to encode negative self-referent words compared to placebo. Lithium was also associated with a lower misclassification rate and lower response bias towards sad facial expressions during the first session. Another significant finding was a higher accuracy of recognising disgust facial expressions in the first visit in those receiving lithium than placebo. During the second visit, there were no significant differences between treatments for all parameters during the second visit, except for a higher accuracy and a lower beta value for fear recognition in lithium than placebo, indicating a bias towards fear responding. We also found statistically meaningful effects on CGT measures of delayed aversion and reward-seeking between treatments in both visits.

Generally, treatment order with carryover, learning and practice effects may contribute to the apparent effects of treatment in a cross-over design study. We used randomisation of order allocation and blinding to help control the impact of treatment order in our study population. Despite this, our findings were clearly affected by order effects, as significant interactions between treatment and order were found in various parameters (except for the ECAT RT) in our study. When found, significant differences appeared to occur in the first session when the tasks were novel.

We doubt that carryover of the effect of lithium treatment on impulsivity and emotional processing was a contributing factor to this, because our participants had at least two weeks’ washout before the second treatment was initiated. Lithium is not metabolized and is primarily excreted by the kidneys. Given that our participants had normal renal functions at screening, we assumed a half-life of 18–36 h. On this basis, lithium would be expected to be eliminated from the body in about a week^26^. However, practice effects could well influence task performance despite our mitigation measure by using different versions of tasks. Also, our findings raise the possibility that the influence of practice might be modified by lithium treatment, as there were no main effects of order in the ANOVA. Additionally, we conducted supplementary VAs. Although significant treatment x order interactions with large effect sizes were observed for some CGT and emotional processing parameters, post-hoc comparisons did not reveal significant treatment differences at either visit. Confidence intervals that included zero in post-hoc tests indicate that the moderate to large effect sizes from the ANOVAs are likely a result of sample size limitation rather than robust treatment effects. These findings were more likely attributable to the small sample size and high between-subject variability relative to the mean differences than to a genuine order effects. Visual inspection of the data did not identify clear outliers.

Other than that, the lack of lithium effects on the CGT and some ETB measures in the current study might be explained by short treatment duration, suboptimal lithium levels (which might be due to adherence issues), inadequate intervals between treatments, small sample size and the absent of current mood disorder. Preclinical studies investigating the effects of lithium on the lab measures of impulsivity typically administered lithium for longer durations, ranging from 3 to 12 weeks^8,10^, although Ohmura and colleagues reported significant reductions in impulsivity after a single dose of lithium in an animal model. Variations in doses (single vs. repeated) and route (intraperitoneal vs. oral) may partially explain the differing findings. Other than adherence issues, variability in lithium levels may also be influenced by factors such as physical activity, body mass index (BMI), and dietary intake. High levels of physical activities and increased BMI have been associated lower lithium levels, whereas dietary intake may contribute to either elevated or reduced lithium levels^27,28^. However, these data were not collected in the present study.

A study involving patients with a recent medically severe suicide attempt reported that six weeks lithium therapy significantly reduced impulsivity (the Immediate Memory Task (IMT) – commission error latency domain)^6^. The interval between assessments of impulsivity in this study was six weeks, while our study intervals were 14–32 days. A shorter between-treatment interval might be a contributing factor for apparent learning and practice effects in our study. The task duration and intensity might be other contributing factors. If the IMT duration (which was not reported in the study) was significantly shorter than the CGT, fatigue effects might contribute to the differing effects of lithium in both studies. Besides, motivation might also be driven by other factors, such as mood and food intake, but these factors were not measured in our study.

The current findings also contradict our previous study investigating the effect of a putative lithium-mimetic agent, ebselen, on the same battery (CGT) in healthy volunteers. We previously reported that ebselen significantly reduced impulsivity as evidenced by decreased delay aversion compared to placebo^19^. The smaller sample size (20 in the ebselen study vs. 16 in the current study) between studies is unlikely to explain the divergent findings. Rather, different outcomes may be attributable to baseline variations in performance, potential ceiling effects of lithium’s neuropsychological effects limiting the detection of improvements in healthy volunteers (observed only for recognition of neutral facial expressions and for encoding of positive and negative self-referent words), dosing (one vs. five days) and pharmacological agents (lithium vs. ebselen).

It is possible that varying effects of lithium and ebselen on two primary molecular targets (inositol monophosphatase (IMPase) and glycogen synthetase kinase-3 β (GSK3β) resulted in different findings. Although both lithium and ebselen are more selective for IMPase than GSK3β, the magnitude of their inhibitions on these enzymes may differ^29^. Further, biological pathways responsible for regulating impulsivity are still unclear. Evidence from a genetic study reported significant associations between single nucleotide polymorphisms in GSK3β and impulsivity in bipolar disorder patients^30^. Effects on glutamatergic neurotransmission may be another contributing factor to the differing effects of lithium and ebselen. Although both drugs can reduce the levels of glutamate-related metabolites in specific brain regions^25,31–33^, their mechanisms of action are different. Ebselen reduces glutamate levels via its inhibitory effects on glutaminase and glutamate dehydrogenase^34^. Acute administration of lithium inhibits the reuptake of glutamate into the presynaptic neurons, while chronic treatment increases glutamate uptake into synaptosomes^35^.

An early positive emotional processing shift induced by acute lithium treatment is consistent with a cognitive neuropsychological model of antidepressant drug action. The model proposes that drugs with antidepressant potential exert a direct effect on emotional processing by producing positive bias early in the treatment before clinical improvement becomes apparent^17^. Acute administration of conventional antidepressants, such as reboxetine and citalopram, was reported to shift emotional processing bias towards more positive biasing in facial expression recognition and emotional encoding and memory in healthy volunteers and unmedicated depressed patients^24,36^. Interestingly, ebselen, a lithium-mimetic agent, produced positive bias in healthy volunteers^19^ but this was not replicated in patients with treatment-resistant depression, potentially due to the effects of current antidepressants used^31,37^. Nevertheless, the findings of the current study in the context of this model corroborate lithium’s effectiveness in the treatment of mood disorders, as evidenced by prolonged reaction time to encode negative self-referent words and a lower misclassification rate and response bias towards sad facial expressions.

A previous meta-analysis evaluating emotional recognition deficits in major depressive disorder reported that the recognition of sad facial expression was preserved in this population, and that the recognition of happy and sad facial expressions was not associated with the medication status (medicated vs. unmedicated)^38^. Our study suggests that lithium’s effectiveness in depression might be attributed to its modulating effects on sadness recognition.

Interestingly, we also found a significantly higher accuracy in recognising disgust facial expressions in the lithium group during the first visit. A similar finding was reported in our previous study in healthy volunteers with ebselen. Notably, ebselen showed the strongest effect in increasing the recognition of disgust facial expressions compared to another significant emotion (happiness)^25^. The exact mechanism for this is unclear. Disgust is generally considered a negative emotion. One possible reason is that disgust is sometimes related to nausea. Although not significant, there was a trend toward higher frequency of nausea in the lithium than in the placebo groups (Fisher’s exact test, *p* = 0.12). From an evolutionary standpoint, disgust is an adaptive emotion that protects humans from potentially contaminated food resources. Randler and colleagues reported that people who were more anxious, disgust-sensitive and susceptible to more negative emotions avoided food more than others after exposure to unpleasant experiences (trout dissection)^39^. However, many of these parameters (trait and state disgust, positive and negative affect schedule) were not evaluated in our study, so inferences cannot be made in relation to disgust-mediated food avoidance.

The effects of lithium on emotional processing was also supported by a previous study assessing the effects of lithium on emotional regulation in healthy volunteers. Artiach Hortelano and colleagues reported that lithium significantly reduced the activation of prefrontal areas during the reappraisal of negatively valenced visual stimuli. Also, there was a greater inverse correlation in functional connectivity between the left amygdala and the bilateral prefrontal cortex in lithium than placebo groups^15^. The outcomes suggest that lithium may regulate emotional responses by reducing activation of the key emotional regulation regions (frontal areas). However, it is important to note that the lithium effects on emotional regulation in that study might differ from the effects of the battery we used in the current study, which focussed primarily on emotion rather than cognition.

Our study has several limitations. Neuropsychological enhancement by lithium is most likely to be observed when there is a scope for improvement. Our participants were healthy volunteers, screened for psychiatric disorders, and thus may have had relatively high baseline performances on some measures. This may explain the limited sensitivity to detect the effects of lithium on some measures used in our study. The observed order effects may reflect the fact that participants performed less well during earlier sessions, providing greater scope for lithium to modulate performance. In later sessions, as performance improved due to practice, the potential for further enhancement was reduced. This suggests that a parallel group design would be a better option. The small sample size reduced the power to detect differences between treatments which may lead to false negative results. Further, the small sample size means that the results regarding between-subject effects are preliminary and must be interpreted cautiously.

The short duration of treatment may not be sufficient to induce substantial changes in other emotional processing domains and measures of decision-making and reward-seeking. Another possibility is the ceiling effects of lithium on certain measures, which could mask the potential effects of lithium. Our study did not perform a stratification of baseline traits. Baseline traits such as impulsivity can moderate neuropsychological responses to medication^40^. Further studies should include larger sample sizes, longer treatment duration, between-subject design, specific clinical populations, a longer interval between study visits to reduce practice effects, and stratification based on baseline traits.

## Conclusions

A short treatment of lithium produced a positive emotional bias by prolonging the response time to encode negative self-referent words and by reducing misclassification and response bias in recognising sad facial expressions, but had no significant effects on impulsivity and reward-seeking. The early positive bias change in emotional processing may contribute to lithium’s effectiveness in the treatment of depression and bipolar disorder.

## Supplementary Information

Below is the link to the electronic supplementary material.

Supplementary Material 1

## Data Availability

The datasets used and/or analysed during the current study are available from the corresponding author on reasonable request.
