# Supplementary material for: Lithium effects on impulsivity and emotional processing
Source: Sci Rep. 2025 Nov 23;15:45216. doi: 10.1038/s41598-025-29216-7 (PMC12749009; doi:10.1038/s41598-025-29216-7)
Supplement: Supplementary file 1 — Supplementary Material 1 [file 41598_2025_29216_MOESM1_ESM.docx]

Table S1. Independent *t*-tests for delay aversion and reward seeking between treatment in each visit

| Parameter | Mean difference | Standard error difference | 95% Confidence Interval (CI) | | t | df | p-value | Cohen’s d & 95%CI |
| --- | --- | --- | --- | --- | --- | --- | --- | --- |
|  |  |  | Lower | Upper |  |  |  |  |
| First visit | | | | | | | |  |
| Delay aversion | 0.114 | 0.096 | -0.091 | 0.318 | 1.188 | 14 | 0.255 | 0.594 (-0.420 – 1.588) |
| Reward seeking | -0.040 | 0.060 | -0.169 | 0.089 | -0.67 | 14 | 0.513 | -0.335 (-1.317 – 0.658) |
| Reward seeking (arcsine transformed) | -0.078 | 0.059 | -0.205 | 0.048 | -1.325 | 14 | 0.103 | -0.662 (-1.661 – 0.358) |
| Second visit | | | | | | | |  |
| Delay aversion | -0.072 | 0.063 | -0.207 | 0.063 | -1.148 | 14 | 0.270 | -0.574 (-1.567 – 0.438) |
| Reward seeking (%) | -0.019 | 0.050 | -0.126 | 0.088 | -0.381 | 14 | 0.709 | -0.190 (-1.169 – 0.796) |
| Reward seeking (arcsine transformed) | -0.058 | 0.053 | -0.172 | 0.057 | -1.082 | 14 | 0.298 | -0.541 (-1.532—0.468) |

Table S2. Independent *t*-tests for the ETB parameters (n=16) between treatments in each visit

| Emotion or valence | t | df | p-value | Mean Difference | Std. Error Difference | 95%CI Lower | 95%CI Upper | Cohen’s d & 95%CI |
| --- | --- | --- | --- | --- | --- | --- | --- | --- |
| FERT accuracy | | | | | | | |  |
| Visit 1 | | | | | | | |  |
| Sad | -1.041 | 14 | 0.315 | -6.250 | 6.002 | -19.123 | 6.623 | -0.521 (-1.510 – 0.487) |
| Fear | 0.772 | 14 | 0.453 | 4.063 | 5.265 | -7.230 | 15.355 | 0.386 (-0.611 – 1.369) |
| Happy | 0.524 | 14 | 0.609 | 1.875 | 3.579 | -5.801 | 9.551 | 0.262 (-0.727 – 1.242) |
| Surprise | 0.697 | 9.52 | 0.502 | 6.875 | 9.864 | -15.254 | 29.004 | 0.348 (-0.646 – 1.331) |
| Disgust | 2.513 | 14 | 0.025* | 10.313 | 4.104 | 1.511 | 19.114 | 1.257 (0.155 – 2.321) |
| Anger | 1.367 | 14 | 0.193 | 5.313 | 3.887 | -3.024 | 13.649 | 0.683 (-0.340 – 1.684) |
| Neutral | 1.106 | 14 | 0.287 | 6.250 | 5.650 | -5.868 | 18.368 | 0.553 (-0.457 – 1.545) |
| Visit 1 (arcsine transformed) | | | | | | | | |
| Sad | -1.056 | 14 | 0.309 | -0.067 | 0.064 | -0.204 | 0.069 | -0.528 (-1.518 – 0.48) |
| Fear | 0.759 | 14 | 0.461 | 0.042 | 0.055 | -0.076 | 0.159 | 0.379 (-0.617 – 1.363) |
| Happy | 0.531 | 14 | 0.604 | 0.022 | 0.042 | -0.068 | 0.113 | 0.266 (-0.724 – 1.246) |
| Surprise | 0.686 | 9.405 | 0.509 | 0.072 | 0.106 | -0.165 | 0.310 | 0.343 (-0.651 – 1.325) |
| Disgust | 2.516 | 14 | 0.025* | 0.105 | 0.042 | 0.015 | 0.194 | 1.258 (0.157 – 2.323) |
| Anger | 1.404 | 14 | 0.182 | 0.057 | 0.041 | -0.030 | 0.145 | 0.702 (-0.323 – 1.704) |
| Neutral | 1.154 | 14 | 0.268 | 0.123 | 0.106 | -0.105 | 0.350 | 0.577 (-0.436 – 1.57) |
| Visit 2 | | | | | | | |  |
| Sad | 0.284 | 14 | 0.781 | 2.500 | 8.801 | -16.376 | 21.376 | 0.142 (-0.842 – 1.121) |
| Fear | 0.815 | 14 | 0.429 | 5.000 | 6.133 | -8.154 | 18.154 | 0.408 (-0.591 – 1.392) |
| Happy | 0.765 | 14 | 0.457 | 2.500 | 3.269 | -4.511 | 9.511 | 0.382 (-0.614 – 1.366) |
| Surprise | -1.127 | 14 | 0.279 | -10.938 | 9.702 | -31.746 | 9.871 | -0.564 (-1.556 – 0.447) |
| Disgust | -0.132 | 14 | 0.897 | -0.938 | 7.086 | -16.135 | 14.260 | -0.066 (-1.045 – 0.915) |
| Anger | -0.776 | 14 | 0.451 | -3.438 | 4.431 | -12.940 | 6.065 | -0.388 (-1.371 – 0.609) |
| Neutral | -0.271 | 14 | 0.790 | -1.250 | 4.605 | -11.127 | 8.627 | -0.136 (-1.115 – 0.848) |
| Visit 2 (arcsine transformed) | | | | | | | | |
| Sad | 0.252 | 14 | 0.805 | 0.023 | 0.091 | -0.172 | 0.218 | 0.126 (-0.857 – 1.105) |
| Fear | 0.703 | 14 | 0.493 | 0.047 | 0.067 | -0.096 | 0.190 | 0.352 (-0.643 – 1.334) |
| Happy | 0.759 | 14 | 0.460 | 0.029 | 0.039 | -0.054 | 0.112 | 0.38 (-0.617 – 1.363) |
| Surprise | -1.134 | 14 | 0.276 | -0.147 | 0.130 | -0.426 | 0.131 | -0.567 (-1.559 – 0.444) |
| Disgust | -0.173 | 14 | 0.865 | -0.013 | 0.076 | -0.176 | 0.150 | -0.087 (-1.066 – 0.895) |
| Anger | -0.786 | 14 | 0.445 | -0.035 | 0.045 | -0.131 | 0.061 | -0.393 (-1.377 – 0.604) |
| Neutral | -0.137 | 14 | 0.893 | -0.015 | 0.106 | -0.241 | 0.212 | -0.069 (-1.048 – 0.913) |
| FERT misclassification | | | | | | | |  |
| Visit 1 | | | | | | | |  |
| Sad | -2.614 | 7.993 | 0.031* | -3.869 | 1.480 | -7.282 | -0.456 | -1.307 (-2.380 – -0.197) |
| Fear | -1.377 | 14 | 0.190 | -1.488 | 1.081 | -3.806 | 0.830 | -0.689 (-1.689 – 0.335) |
| Happy | 0.095 | 14 | 0.925 | 0.060 | 0.625 | -1.280 | 1.399 | 0.048 (-0.933 – 1.027) |
| Surprise | 0.168 | 14 | 0.869 | 0.238 | 1.415 | -2.798 | 3.274 | 0.084 (-0.898 – 1.063) |
| Anger | -0.673 | 14 | 0.512 | -0.714 | 1.061 | -2.990 | 1.561 | -0.337 (-1.318 – 0.657) |
| Disgust | -0.388 | 14 | 0.704 | -0.476 | 1.227 | -3.108 | 2.155 | -0.194 (-1.173 – 0.792) |
| Neutral | 0.563 | 14 | 0.582 | 1.510 | 2.683 | -4.244 | 7.265 | 0.281 (-0.709 – 1.262) |
| Visit 1 (arcsine transformed) | | | | | | | | |
| Sad | -2.204 | 14 | 0.045* | -0.072 | 0.033 | -0.142 | -0.002 | -1.102 (-2.146 – -0.025) |
| Fear | -1.397 | 14 | 0.184 | -0.048 | 0.034 | -0.122 | 0.026 | -0.699 (-1.7 – 0.326) |
| Happy | -0.302 | 14 | 0.767 | -0.009 | 0.030 | -0.074 | 0.056 | -0.151 (-1.13 – 0.833) |
| Surprise | 0.27 | 14 | 0.791 | 0.010 | 0.035 | -0.067 | 0.086 | 0.135 (-0.849 – 1.114) |
| Disgust | -0.374 | 14 | 0.714 | -0.013 | 0.034 | -0.085 | 0.060 | -0.187 (-1.166 – 0.799) |
| Anger | -0.685 | 14 | 0.504 | -0.021 | 0.030 | -0.086 | 0.044 | -0.343 (-1.325 – 0.651) |
| Neutral | 0.558 | 14 | 0.586 | 0.018 | 0.033 | -0.052 | 0.089 | 0.279 (-0.711 – 1.259) |
| Visit 2 |  |  |  |  |  |  |  |  |
| Sad | 1.455 | 14 | 0.168 | 1.786 | 1.227 | -0.846 | 4.417 | 0.728 (-0.300 – 1.731) |
| Fear | 2.008 | 14 | 0.064 | 3.095 | 1.542 | -0.211 | 6.402 | 1.004 (-0.059 – 2.035) |
| Happy | 1.271 | 9.923 | 0.233 | 0.179 | 0.140 | -0.135 | 0.492 | 0.635 (-0.382 – 1.632) |
| Surprise | -1.148 | 14 | 0.270 | -1.786 | 1.555 | -5.122 | 1.550 | -0.574 (-1.567 – 0.438) |
| Anger | 0.114 | 14 | 0.911 | 0.119 | 1.045 | -2.123 | 2.361 | 0.057 (-0.924 – 1.036) |
| Disgust | 0.727 | 14 | 0.479 | 0.595 | 0.819 | -1.161 | 2.351 | 0.364 (-0.632 – 1.346) |
| Neutral | -0.971 | 14 | 0.348 | -2.552 | 2.627 | -8.188 | 3.083 | -0.486 (-1.473 – 0.519) |
| Visit 2 (arcsine transformed) | | | | | | | | |
| Sad | 1.443 | 14 | 0.171 | 0.057 | 0.040 | -0.028 | 0.142 | 0.722 (-0.305 – 1.725) |
| Fear | 2.512 | 14 | 0.025* | 0.101 | 0.040 | 0.015 | 0.186 | 1.256 (0.155 – 2.321) |
| Happy | 1.222 | 14 | 0.242 | 0.021 | 0.017 | -0.016 | 0.057 | 0.611 (-0.405 – 1.606) |
| Surprise | -1.16 | 14 | 0.265 | -0.052 | 0.045 | -0.147 | 0.044 | -0.58 (-1.573 – 0.433) |
| Disgust | 0.525 | 14 | 0.608 | 0.012 | 0.022 | -0.036 | 0.059 | 0.262 (-0.727 – 1.242) |
| Anger | 0.052 | 14 | 0.959 | 0.001 | 0.025 | -0.053 | 0.056 | 0.026 (-0.954 – 1.006) |
| Neutral | -1.002 | 14 | 0.333 | -0.029 | 0.029 | -0.092 | 0.033 | -0.501 (-1.489 – 0.505) |
| FERT RT | | | | | | | | |
| Visit 1 | | | | | | | | |
| Sad | -0.294 | 9.044 | 0.775 | -0.120 | 0.407 | -1.039 | 0.800 | -0.147 (-1.126 – 0.837) |
| Fear | 0.009 | 14 | 0.993 | 0.003 | 0.377 | -0.804 | 0.811 | 0.004 (-0.976 – 0.984) |
| Happy | -0.696 | 14 | 0.498 | -0.175 | 0.252 | -0.715 | 0.365 | -0.348 (-1.330 – 0.646) |
| Surprise | -0.561 | 14 | 0.584 | -0.181 | 0.323 | -0.875 | 0.512 | -0.280 (-1.261 – 0.710) |
| Disgust | -0.445 | 14 | 0.663 | -0.082 | 0.184 | -0.476 | 0.313 | -0.222 (-1.202 – 0.765) |
| Anger | -0.432 | 14 | 0.672 | -0.138 | 0.319 | -0.821 | 0.546 | -0.216 (-1.195 – 0.771) |
| Neutral | -0.887 | 14 | 0.390 | -0.149 | 0.168 | -0.510 | 0.212 | -0.443 (-1.429 – 0.558) |
| Visit 2 |  |  |  |  |  |  |  |  |
| Sad | 1.79 | 14 | 0.095 | 0.408 | 0.228 | -0.081 | 0.898 | 0.895 (-0.153 – 1.914) |
| Fear | 1.33 | 14 | 0.205 | 0.493 | 0.371 | -0.302 | 1.288 | 0.665 (-0.356 – 1.664) |
| Happy | 1.303 | 14 | 0.214 | 0.317 | 0.244 | -0.205 | 0.840 | 0.651 (-0.368 – 1.649) |
| Surprise | 1.876 | 14 | 0.082 | 0.509 | 0.272 | -0.073 | 1.092 | 0.938 (-0.115 – 1.962) |
| Disgust | 1.829 | 14 | 0.089 | 0.526 | 0.287 | -0.091 | 1.142 | 0.914 (-0.136 – 1.936) |
| Anger | 1.326 | 14 | 0.206 | 0.381 | 0.287 | -0.235 | 0.998 | 0.663 (-0.358 – 1.662) |
| Neutral | 0.036 | 14 | 0.972 | 0.015 | 0.410 | -0.864 | 0.893 | 0.018 (-0.962 – 0.998) |
| FERT D prime | | | | | | | |  |
| Visit 1 | | | | | | | |  |
| Sad | -0.18 | 14 | 0.860 | -0.002 | 0.013 | -0.031 | 0.026 | -0.09 (-1.069 – 0.892) |
| Fear | 1.327 | 14 | 0.206 | 0.024 | 0.018 | -0.015 | 0.063 | 0.663 (-0.358 – 1.662) |
| Happy | 0.47 | 14 | 0.646 | 0.005 | 0.010 | -0.016 | 0.025 | 0.235 (-0.753 – 1.214) |
| Surprise | 0.75 | 9.342 | 0.472 | 0.018 | 0.024 | -0.036 | 0.072 | 0.375 (-0.621 – 1.358) |
| Disgust | 2.078 | 14 | 0.057 | 0.034 | 0.016 | -0.001 | 0.069 | 1.039 (-0.028 – 2.075) |
| Anger | 1.613 | 14 | 0.129 | 0.020 | 0.012 | -0.006 | 0.046 | 0.807 (-0.23 – 1.817) |
| Neutral | 0.981 | 14 | 0.343 | 0.018 | 0.018 | -0.021 | 0.056 | 0.491 (-0.514 – 1.479) |
| Visit 2 | | | | | | | |  |
| Sad | -0.084 | 14 | 0.934 | -0.002 | 0.024 | -0.054 | 0.050 | -0.042 (-1.021 – 0.939) |
| Fear | -0.677 | 14 | 0.509 | -0.015 | 0.022 | -0.061 | 0.032 | -0.339 (-1.321 – 0.655) |
| Happy | 0.691 | 14 | 0.501 | 0.006 | 0.008 | -0.012 | 0.023 | 0.346 (-0.649 – 1.328) |
| Surprise | -0.3 | 13 | 0.769 | -0.005 | 0.015 | -0.037 | 0.028 | -0.156 (-1.169 – 0.864) |
| Disgust | -0.323 | 14 | 0.751 | -0.008 | 0.023 | -0.057 | 0.042 | -0.162 (-1.14 – 0.823) |
| Anger | -0.87 | 14 | 0.399 | -0.012 | 0.014 | -0.041 | 0.017 | -0.435 (-1.42 – 0.565) |
| Neutral | 0.204 | 14 | 0.841 | 0.004 | 0.019 | -0.037 | 0.045 | 0.102 (-0.881 – 1.081) |
| FERT beta | | | | | | | |  |
| Visit 1 | | | | | | | |  |
| Sad | 2.241 | 9.061 | 0.052 | 0.213 | 0.095 | -0.002 | 0.428 | 1.121 (0.041– 2.166) |
| Fear | 1.386 | 14 | 0.187 | 0.088 | 0.064 | -0.048 | 0.225 | 0.693 (-0.331 – 1.694) |
| Happy | -0.121 | 14 | 0.906 | -0.007 | 0.054 | -0.122 | 0.109 | -0.06 (-1.039 – 0.921) |
| Surprise | 0.099 | 14 | 0.923 | 0.009 | 0.094 | -0.193 | 0.211 | 0.049 (-0.932 – 1.029) |
| Disgust | 0.438 | 14 | 0.668 | 0.030 | 0.068 | -0.117 | 0.177 | 0.219 (-0.768 – 1.198) |
| Anger | 0.246 | 14 | 0.809 | 0.017 | 0.068 | -0.129 | 0.162 | 0.123 (-0.86 – 1.102) |
| Neutral | -1.076 | 14 | 0.300 | -0.242 | 0.225 | -0.725 | 0.241 | -0.538 (-1.528 – 0.471) |
| Visit 2 | | | | | | | |  |
| Sad | -1.402 | 14 | 0.183 | -0.111 | 0.079 | -0.280 | 0.059 | -0.701 (-1.703 – 0.324) |
| Fear | -2.576 | 14 | 0.022* | -0.203 | 0.079 | -0.372 | -0.034 | -1.288 (-2.357 – -0.181) |
| Happy | -1.237 | 14 | 0.237 | -0.025 | 0.020 | -0.069 | 0.018 | -0.618 (-1.614 – 0.398) |
| Surprise | 0.913 | 13 | 0.378 | 0.084 | 0.092 | -0.115 | 0.284 | 0.472 (-0.566 – 1.494) |
| Disgust | -0.663 | 14 | 0.518 | -0.033 | 0.049 | -0.138 | 0.073 | -0.332 (-1.313 – 0.662) |
| Anger | 0.093 | 14 | 0.927 | 0.005 | 0.058 | -0.119 | 0.130 | 0.046 (-0.934 – 1.026) |
| Neutral | 0.249 | 14 | 0.807 | 0.051 | 0.205 | -0.388 | 0.490 | 0.125 (-0.859 – 1.103) |
| ECAT | | | | | | | |  |
| Accuracy | | | | | | | |  |
| Visit 1 | | | | | | | |  |
| Positive | -0.154 | 14 | 0.88 | -0.625 | 4.057 | -9.327 | 8.077 | -0.077 (-1.056 – 0.905) |
| Negative | -0.683 | 14 | 0.506 | -1.875 | 2.745 | -7.762 | 4.012 | -0.342 (-1.324 – 0.652) |
| Visit 2 | | | | | | | | |
| Positive | 0.231 | 14 | 0.821 | 0.625 | 2.704 | -5.174 | 6.424 | 0.116 (-0.867 – 1.094) |
| Negative | -0.782 | 14 | 0.447 | -1.875 | 2.397 | -7.017 | 3.267 | -0.391 (-1.375 – 0.606) |
| RT | | | | | | | | |
| Visit 1 | | | | | | | | |
| Positive | 0.382 | 14 | 0.708 | 0.048 | 0.127 | -0.224 | 0.320 | 0.191 (-0.795 – 1.170) |
| Negative | 0.472 | 14 | 0.644 | 0.067 | 0.142 | -0.237 | 0.371 | 0.236 (-0.752 – 1.216) |
| Visit 2 |  |  |  |  |  |  |  |  |
| Positive | 1.138 | 9.9 | 0.282 | 0.117 | 0.103 | -0.113 | 0.347 | 0.569 (-0.443 – 1.561) |
| Negative | 1.664 | 10.5 | 0.126 | 0.264 | 0.159 | -0.087 | 0.616 | 0.832 (-0.208 – 1.845) |
| EREC | | | | | | | |  |
| Accuracy | | | | | | | |  |
| Visit 1 | | | | | | | |  |
| Positive | 0.714 | 14 | 0.487 | 0.625 | 0.875 | -1.252 | 2.502 | 0.357 (-0.638 – 1.340) |
| Negative | 0.732 | 14 | 0.477 | 0.625 | 0.854 | -1.207 | 2.457 | 0.366 (-0.630 – 1.349) |
| Visit 2 | | | | | | | |  |
| Positive | 0.113 | 14 | 0.912 | 0.125 | 1.109 | -2.254 | 2.504 | 0.056 (-0.925 – 1.036) |
| Negative | 0 | 14 | 1.000 | 0.000 | 0.973 | -2.087 | 2.087 | 0.000 (-0.980 – 0.980) |
| Misclassification | | | | | | | | |
| Visit 1 | | | | | | | | |
| Positive | -1.738 | 14 | 0.104 | -1.75 | 1.007 | -3.909 | 0.409 | -0.869 (-1.886 – 0.175) |
| Negative | -2.366 | 7 | 0.05 | -2 | 0.845 | -3.998 | -0.002 | -1.183 (-2.238 – 0.094) |
| Visit 2 | | | | | | | | |
| Positive | 1.883 | 7.933 | 0.097 | 1.375 | 0.73 | -0.312 | 3.062 | 0.941 (-0.113 – 1.966) |
| Negative | 0.552 | 14 | 0.59 | 0.375 | 0.68 | -1.083 | 1.833 | 0.276 (-0.714 – 1.256) |
| EMEM | | | | | | | | |
| Accuracy | | | | | | | | |
| Visit 1 | | | | | | | | |
| Positive | 0.879 | 14 | 0.394 | 0.040 | 0.045 | -0.058 | 0.138 | 0.439 (-0.561 – 1.425) |
| Negative | -1.24 | 14 | 0.235 | -0.043 | 0.035 | -0.117 | 0.031 | -0.62 (-1.615 – 0.397) |
| Visit 2 | | | | | | | | |
| Positive | 0.253 | 14 | 0.804 | 0.010 | 0.041 | -0.077 | 0.098 | 0.126 (-0.857 – 1.105) |
| Negative | 0.025 | 14 | 0.980 | 0.001 | 0.043 | -0.091 | 0.094 | 0.013 (-0.968 – 0.992) |
| RT | | | | | | | | |
| Visit 1 | | | | | | | | |
| Positive | -0.150 | 14 | 0.883 | -0.016 | 0.109 | -0.250 | 0.218 | -0.075 (-1.054 – 0.907) |
| Negative | -0.388 | 14 | 0.704 | -0.050 | 0.128 | -0.323 | 0.224 | -0.194 (-1.173 – 0.792) |
| Visit 2 | | | | | | | | |
| Positive | 0.399 | 14 | 0.696 | 0.047 | 0.119 | -0.208 | 0.303 | 0.199 (-0.787 – 1.178) |
| Negative | 0.502 | 14 | 0.623 | 0.069 | 0.137 | -0.226 | 0.364 | 0.251 (-0.738 – 1.231) |
| Misclassification | | | | | | | | |
| Visit 1 | | | | | | | | |
| Positive | -0.879 | 14 | 0.394 | -0.040 | 0.045 | -0.138 | 0.058 | -0.439 (-1.425 – 0.561) |
| Negative | 1.24 | 14 | 0.235 | 0.043 | 0.035 | -0.031 | 0.117 | 0.62 (-0.397 – 1.615) |
| Visit 2 | | | | | | | | |
| Positive | -0.253 | 14 | 0.804 | -0.010 | 0.041 | -0.098 | 0.077 | -0.126 (-1.105 – 0.857) |
| Negative | -0.025 | 14 | 0.980 | -0.001 | 0.043 | -0.094 | 0.091 | -0.013 (-0.992 – 0.968) |
| D Prime | | | | | | | | |
| Visit 1 | | | | | | | | |
| Positive | 0.652 | 14 | 0.525 | 0.016 | 0.025 | -0.037 | 0.069 | 0.326 (-0.667 – 1.308) |
| Negative | -1.452 | 14 | 0.169 | -0.025 | 0.018 | -0.063 | 0.012 | -0.726 (-1.729 – 0.302) |
| Visit 2 | | | | | | | | |
| Positive | 0.047 | 14 | 0.963 | 0.001 | 0.018 | -0.037 | 0.039 | 0.024 (-0.957 – 1.003) |
| Negative | 0.187 | 14 | 0.855 | 0.004 | 0.022 | -0.043 | 0.051 | 0.093 (-0.889 – 1.072) |
| FDOT | | | | | | | | |
| Visit 1 | | | | | | | | |
| Unmasked | | | | | | | | |
| Happy | -1.011 | 14 | 0.329 | -0.020 | 0.020 | -0.064 | 0.023 | -0.506 (-1.494 – 0.5) |
| Fear | -0.35 | 14 | 0.732 | -0.007 | 0.019 | -0.048 | 0.034 | -0.175 (-1.154 – 0.81) |
| Masked | | | | | | | | |
| Happy | -0.848 | 14 | 0.411 | -0.019 | 0.022 | -0.067 | 0.029 | -0.424 (-1.409 – 0.576) |
| Fear | 0.331 | 14 | 0.745 | 0.008 | 0.025 | -0.045 | 0.061 | 0.166 (-0.819 – 1.145) |
| Visit 2 | | | | | | | | |
| Unmasked | | | | | | | | |
| Happy | 0.079 | 14 | 0.938 | 0.002 | 0.023 | -0.048 | 0.052 | 0.04 (-0.941 – 1.019) |
| Fear | 1.212 | 14 | 0.246 | 0.032 | 0.026 | -0.024 | 0.088 | 0.606 (-0.409 – 1.601) |
| Masked | | | | | | | | |
| Happy | 0.962 | 14 | 0.352 | 0.031 | 0.032 | -0.038 | 0.100 | 0.481 (-0.523 – 1.469) |
| Fear | 0.134 | 14 | 0.896 | 0.004 | 0.032 | -0.064 | 0.072 | 0.067 (-0.915 – 1.046) |

Table S3. Three-way and two-way ANOVAs for the ECAT (accuracy), EREC (misclassification), EMEM (accuracy, RT, misclassification, d prime, and beta) and FDOT (attentional vigilance score).

| Parameter | df | Mean Square | F | p-value | Partial Eta Squared |
| --- | --- | --- | --- | --- | --- |
| FERT | | | | | |
| RT | | | | | |
| Treatment * Order | 1 |  | 3.81 | 0.071 | 0.214 |
| Error | 14 |  |  |  |  |
| Emotion * Order | 6 |  | 0.575 | 0.750 | 0.039 |
| Error (Emotion) | 84 |  |  |  |  |
| Treatment * Emotion | 6 |  | 1.195 | 0.317 | 0.079 |
| Treatment * Emotion * Order | 6 |  | 1.254 | 0.288 | 0.082 |
| Error (Treatment * Emotion) | 84 |  |  |  |  |
| ECAT | | | | | |
| Accuracy | | | | | |
| Treatment * Order | 1 | 14.062 | 0.674 | 0.425 | 0.046 |
| Error (Treatment) | 14 | 20.871 |  |  |  |
| Valence * Order | 1 | 1.563 | 0.057 | 0.815 | 0.004 |
| Error (Valence) | 14 | 27.567 |  |  |  |
| Treatment * Valence | 1 | 6.25 | 0.327 | 0.576 | 0.023 |
| Treatment * Valence * Order | 1 | 14.062 | 0.737 | 0.405 | 0.05 |
| Error (Treatment*Valence) | 14 | 19.085 |  |  |  |
| EREC | | | | | |
| Misclassification | | | | | |
| treatment * Order | 1 | 0.016 | 0.024 | 0.879 | 0.002 |
| Error(treatment) | 14 | 0.650 |  |  |  |
| valence * Order | 1 | 0.141 | 0.236 | 0.635 | 0.017 |
| Error(valence) | 14 | 0.596 |  |  |  |
| treatment * valence | 1 | 0.766 | 1.036 | 0.326 | 0.069 |
| treatment * valence * Order | 1 | 0.141 | 0.190 | 0.669 | 0.013 |
| Error(treatment*valence) | 14 | 0.739 |  |  |  |
| FDOT | | | | | |
| Attentional vigilance score | | | | | |
| treatment * Order | 1 | 0.002 | 0.44 | 0.518 | 0.03 |
| Error(treatment) | 14 | 0.005 |  |  |  |
| valance * Order | 1 | 0.001 | 0.187 | 0.672 | 0.013 |
| Error(valance) | 14 | 0.004 |  |  |  |
| condition * Order | 1 | 0 | 0.085 | 0.775 | 0.006 |
| Error(condition) | 14 | 0.001 |  |  |  |
| treatment * valance | 1 | 0.001 | 0.475 | 0.502 | 0.033 |
| treatment * valance * Order | 1 | 0.005 | 2.496 | 0.136 | 0.151 |
| Error(treatment*valance) | 14 | 0.002 |  |  |  |
| treatment * condition | 1 | 0 | 0.128 | 0.726 | 0.009 |
| treatment * condition * Order | 1 | 0.002 | 1.754 | 0.207 | 0.111 |
| Error(treatment*condition) | 14 | 0.001 |  |  |  |
| valance * condition | 1 | 0.001 | 0.424 | 0.525 | 0.029 |
| valance * condition * Order | 1 | 0.002 | 1.418 | 0.254 | 0.092 |
| Error(valance*condition) | 14 | 0.002 |  |  |  |
| treatment * valance * condition | 1 | 0.001 | 0.426 | 0.525 | 0.03 |
| treatment * valance * condition * Order | 1 | 0.01 | 4.563 | 0.051 | 0.246 |
| Error(treatment*valance*condition) | 14 | 0.002 |  |  |  |
| EMEM | | | | | |
| Accuracy | | | | | |
| treatment * Order | 1 | 49.171 | 2.438 | 0.141 | 0.148 |
| Error(treatment) | 14 | 20.17 |  |  |  |
| valence * Order | 1 | 33.555 | 0.923 | 0.353 | 0.062 |
| Error(valence) | 14 | 36.37 |  |  |  |
| treatment * valence | 1 | 33.555 | 1.303 | 0.273 | 0.085 |
| treatment * valence * Order | 1 | 20.914 | 0.812 | 0.383 | 0.055 |
| Error(treatment*valence) | 14 | 25.747 |  |  |  |
| RT | | | | | |
| treatment * Order | 1 | 0.025 | 1.281 | 0.277 | 0.084 |
| Error(treatment) | 14 | 0.019 |  |  |  |
| valence * Order | 1 | 0.003 | 0.75 | 0.401 | 0.051 |
| Error(valence) | 14 | 0.004 |  |  |  |
| treatment * valence | 1 | 0 | 0.026 | 0.873 | 0.002 |
| treatment * valence * Order | 1 | 0.015 | 2.988 | 0.106 | 0.176 |
| Error(treatment*valence) | 14 | 0.005 |  |  |  |
| Misclassification | | | | | |
| treatment * Order | 1 | 49.171 | 2.438 | 0.141 | 0.148 |
| Error(treatment) | 14 | 20.17 |  |  |  |
| valence * Order | 1 | 33.555 | 0.923 | 0.353 | 0.062 |
| Error(valence) | 14 | 36.37 |  |  |  |
| treatment * valence | 1 | 33.555 | 1.303 | 0.273 | 0.085 |
| treatment * valence * Order | 1 | 20.914 | 0.812 | 0.383 | 0.055 |
| Error(treatment*valence) | 14 | 25.747 |  |  |  |
| D prime | | | | | |
| treatment * Order | 1 | 0.009 | 2.15 | 0.165 | 0.133 |
| Error(treatment) | 14 | 0.004 |  |  |  |
| valence * Order | 1 | 0.004 | 0.684 | 0.422 | 0.047 |
| Error(valence) | 14 | 0.006 |  |  |  |
| treatment * valence | 1 | 0.008 | 1.964 | 0.183 | 0.123 |
| treatment * valence * Order | 1 | 0.003 | 0.646 | 0.435 | 0.044 |
| Error(treatment*valence) | 14 | 0.004 |  |  |  |
| Beta | | | | | |
| treatment * Order | 1 | 0 | 2.536 | 0.134 | 0.153 |
| Error(treatment) | 14 | 0 |  |  |  |
| valence * Order | 1 | 0 | 1.033 | 0.327 | 0.069 |
| Error(valence) | 14 | 0 |  |  |  |
| treatment * valence | 1 | 0 | 1.062 | 0.32 | 0.071 |
| treatment * valence * Order | 1 | 0 | 0.88 | 0.364 | 0.059 |
| Error(treatment*valence) | 14 | 0 |  |  |  |

Table S4. Three-way and two-way ANOVAs for the FERT (accuracy, misclassification), ECAT (accuracy), EMEM (accuracy, misclassification) (arcsine transformed).

| Parameter | df | Mean Square | F | p-value | Partial Eta Squared |
| --- | --- | --- | --- | --- | --- |
| FERT | | | | | |
| Accuracy | | | | | |
| Treatment * Order | 1 | 0.020 | 1.769 | 0.205 | 0.112 |
| Error (Treatment) | 14 | 0.012 |  |  |  |
| Emotion * Order | 2.6 | 0.051 | 0.606 | 0.595 | 0.042 |
| Error (Emotion) | 37.0 | 0.084 |  |  |  |
| Treatment * Emotion | 3.0 | 0.020 | 1.041 | 0.384 | 0.069 |
| Treatment * Emotion * Order | 3.0 | 0.131 | 6.670 | <0.001* | 0.323 |
| Error (Treatment * Emotion) | 41.3 | 0.020 |  |  |  |
| Misclassification | | | | | |
| Treatment * Order | 1 | 0.000 | 0.412 | 0.531 | 0.029 |
| Error (Treatment) | 14 | 0.001 |  |  |  |
| Emotion * Order | 6 | 0.013 | 1.748 | 0.120 | 0.111 |
| Error (Emotion) | 84 | 0.007 |  |  |  |
| Treatment * Emotion | 6 | 0.002 | 0.990 | 0.438 | 0.066 |
| Treatment * Emotion * Order | 6 | 0.015 | 8.432 | <0.001* | 0.376 |
| Error (Treatment * Emotion) | 84 | 0.002 |  |  |  |
| ECAT | | | | | |
| Accuracy | | | | | |
| Treatment * Order | 1 | 0.003 | 0.220 | 0.646 | 0.015 |
| Error (Treatment) | 14 | 0.012 |  |  |  |
| Valence * Order | 1 | 0.000 | 0.008 | 0.932 | 0.001 |
| Error (Valence) | 14 | 0.023 |  |  |  |
| Treatment * Valence | 1 | 0.005 | 0.320 | 0.581 | 0.022 |
| Treatment * Valence * Order | 1 | 0.012 | 0.801 | 0.386 | 0.054 |
| Error (Treatment*Valence) | 14 | 0.015 |  |  |  |
| EMEM | | | | | |
| Accuracy | | | | | |
| treatment * Order | 1 | 0.010 | 2.276 | 0.154 | 0.140 |
| Error(treatment) | 14 | 0.004 |  |  |  |
| valence * Order | 1 | 0.005 | 0.759 | 0.398 | 0.051 |
| Error(valence) | 14 | 0.007 |  |  |  |
| treatment * valence | 1 | 0.008 | 1.749 | 0.207 | 0.111 |
| treatment * valence * Order | 1 | 0.004 | 0.730 | 0.407 | 0.050 |
| Error(treatment*valence) | 14 | 0.005 |  |  |  |
| Misclassification | | | | | |
| treatment * Order | 1 | 0.010 | 2.276 | 0.154 | 0.140 |
| Error(treatment) | 14 | 0.004 |  |  |  |
| valence * Order | 1 | 0.005 | 0.759 | 0.398 | 0.051 |
| Error(valence) | 14 | 0.007 |  |  |  |
| treatment * valence | 1 | 0.008 | 1.749 | 0.207 | 0.111 |
| treatment * valence * Order | 1 | 0.004 | 0.730 | 0.407 | 0.050 |
| Error(treatment*valence) | 14 | 0.005 |  |  |  |

Table S5. Independent *t*-tests for the ETB parameters (n=16) between treatments in each visit (arcsine transformed)

| Emotion or Valence | t | df | p-value | Mean Difference | Std. Error Difference | 95%CI Lower | 95%CI Upper | Cohen’s d & 95%CI |
| --- | --- | --- | --- | --- | --- | --- | --- | --- |
| FERT | | | | | | | | |
| Accuracy | | | | | | | | |
| Visit 1 | | | | | | | | |
| Sad | -1.056 | 14 | 0.309 | -0.067 | 0.064 | -0.204 | 0.069 | -0.528 (-1.518 – 0.48) |
| Fear | 0.759 | 14 | 0.461 | 0.042 | 0.055 | -0.076 | 0.159 | 0.379 (-0.617 – 1.363) |
| Happy | 0.531 | 14 | 0.604 | 0.022 | 0.042 | -0.068 | 0.113 | 0.266 (-0.724 – 1.246) |
| Surprise | 0.686 | 9.405 | 0.509 | 0.072 | 0.106 | -0.165 | 0.310 | 0.343 (-0.651 – 1.325) |
| Disgust | 2.516 | 14 | 0.025* | 0.105 | 0.042 | 0.015 | 0.194 | 1.258 (0.157 – 2.323) |
| Anger | 1.404 | 14 | 0.182 | 0.057 | 0.041 | -0.030 | 0.145 | 0.702 (-0.323 – 1.704) |
| Neutral | 1.154 | 14 | 0.268 | 0.123 | 0.106 | -0.105 | 0.350 | 0.577 (-0.436 – 1.57) |
| Visit 2 | | | | | | | |  |
| Sad | 0.252 | 14 | 0.805 | 0.023 | 0.091 | -0.172 | 0.218 | 0.126 (-0.857 – 1.105) |
| Fear | 0.703 | 14 | 0.493 | 0.047 | 0.067 | -0.096 | 0.190 | 0.352 (-0.643 – 1.334) |
| Happy | 0.759 | 14 | 0.460 | 0.029 | 0.039 | -0.054 | 0.112 | 0.38 (-0.617 – 1.363) |
| Surprise | -1.134 | 14 | 0.276 | -0.147 | 0.130 | -0.426 | 0.131 | -0.567 (-1.559 – 0.444) |
| Disgust | -0.173 | 14 | 0.865 | -0.013 | 0.076 | -0.176 | 0.150 | -0.087 (-1.066 – 0.895) |
| Anger | -0.786 | 14 | 0.445 | -0.035 | 0.045 | -0.131 | 0.061 | -0.393 (-1.377 – 0.604) |
| Neutral | -0.137 | 14 | 0.893 | -0.015 | 0.106 | -0.241 | 0.212 | -0.069 (-1.048 – 0.913) |
| Misclassification | | | | | | | | |
| Visit 1 | | | | | | | | |
| Sad | -2.204 | 14 | 0.045* | -0.072 | 0.033 | -0.142 | -0.002 | -1.102 (-2.146 – -0.025) |
| Fear | -1.397 | 14 | 0.184 | -0.048 | 0.034 | -0.122 | 0.026 | -0.699 (-1.7 – 0.326) |
| Happy | -0.302 | 14 | 0.767 | -0.009 | 0.030 | -0.074 | 0.056 | -0.151 (-1.13 – 0.833) |
| Surprise | 0.27 | 14 | 0.791 | 0.010 | 0.035 | -0.067 | 0.086 | 0.135 (-0.849 – 1.114) |
| Disgust | -0.374 | 14 | 0.714 | -0.013 | 0.034 | -0.085 | 0.060 | -0.187 (-1.166 – 0.799) |
| Anger | -0.685 | 14 | 0.504 | -0.021 | 0.030 | -0.086 | 0.044 | -0.343 (-1.325 – 0.651) |
| Neutral | 0.558 | 14 | 0.586 | 0.018 | 0.033 | -0.052 | 0.089 | 0.279 (-0.711 – 1.259) |
| Visit 2 | | | | | | | | |
| Sad | 1.443 | 14 | 0.171 | 0.057 | 0.040 | -0.028 | 0.142 | 0.722 (-0.305 – 1.725) |
| Fear | 2.512 | 14 | 0.025* | 0.101 | 0.040 | 0.015 | 0.186 | 1.256 (0.155 – 2.321) |
| Happy | 1.222 | 14 | 0.242 | 0.021 | 0.017 | -0.016 | 0.057 | 0.611 (-0.405 – 1.606) |
| Surprise | -1.16 | 14 | 0.265 | -0.052 | 0.045 | -0.147 | 0.044 | -0.58 (-1.573 – 0.433) |
| Disgust | 0.525 | 14 | 0.608 | 0.012 | 0.022 | -0.036 | 0.059 | 0.262 (-0.727 – 1.242) |
| Anger | 0.052 | 14 | 0.959 | 0.001 | 0.025 | -0.053 | 0.056 | 0.026 (-0.954 – 1.006) |
| Neutral | -1.002 | 14 | 0.333 | -0.029 | 0.029 | -0.092 | 0.033 | -0.501 (-1.489 – 0.505) |
| ECAT | | | | | | | | |
| Accuracy | | | | | | | | |
| Visit 1 |  |  |  |  |  |  |  |  |
| Positive | 0.192 | 14 | 0.850 | 0.017 | 0.091 | -0.177 | 0.212 | 0.096 (-0.886 – 1.075) |
| Negative | -0.383 | 14 | 0.708 | -0.031 | 0.081 | -0.205 | 0.143 | -0.191 (-1.171 – 0.794) |
| Visit 2 | | | | | | | | |
| Positive | 0.15 | 14 | 0.883 | 0.0120 | 0.080 | -0.160 | 0.184 | 0.075 (-0.907 – 1.054) |
| Negative | -0.655 | 14 | 0.523 | -0.050 | 0.076 | -0.213 | 0.113 | -0.327 (-1.309 – 0.666) |
| EMEM | | | | | | | | |
| Accuracy | | | | | | | | |
| Visit 1 | | | | | | | | |
| Positive |  |  |  |  |  |  |  |  |
| Negative |  |  |  |  |  |  |  |  |
| Visit 2 | | | | | | | | |
| Positive |  |  |  |  |  |  |  |  |
| Negative |  |  |  |  |  |  |  |  |


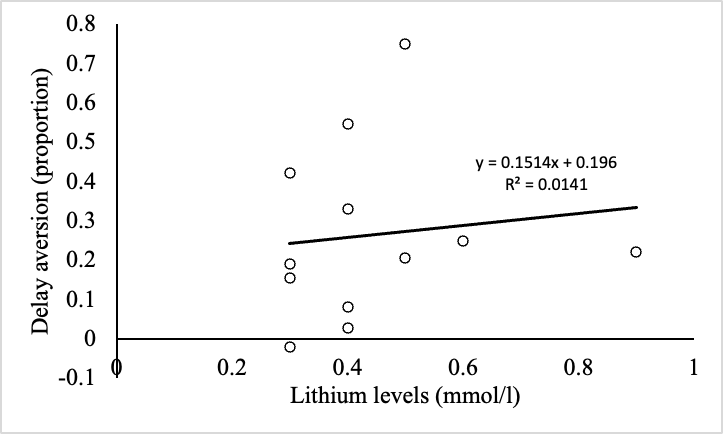


Figure S1. Correlation between lithium levels and delay aversion.
